# Role of antenatal and postnatal care in contraceptive use during postpartum period in western Ethiopia: a cross sectional study

**DOI:** 10.1186/s13104-018-3698-6

**Published:** 2018-08-13

**Authors:** Takele Teshome Teka, Tesfaye Regassa Feyissa, Alemu Sufa Melka, Firew Tekle Bobo

**Affiliations:** 1 EngenderHealth Ethiopia, PoBOX156, 1110 Addis Ababa, Ethiopia; 2 Department of Public Health, College of Medical and Health sciences, Wollega University, Po BOX 395, Nekemte, Ethiopia

**Keywords:** Contraception, Postpartum, ANC, Ethiopia, PNC, Family planning

## Abstract

**Objective:**

Little has been known about the magnitude and predictors of contraceptive use in extended postpartum period in Ethiopia. Thus, this study aims to assess the magnitude and determinants of contraception utilization in extended postpartum period. A community based cross-sectional survey was conducted in Gida Ayana district, Oromia regional state, Ethiopia in February 2015. Six hundred and three postpartum women were included using a multistage sampling technique. Descriptive statistics were used to summarize the data and logistic regressions were used to assess the predictors of modern family planning use at 95% confidence interval.

**Results:**

The proportion of women using any of the modern family planning in extended postpartum period was 45.4%. Women who had four and more antenatal care visits (AOR = 2.93; 95% CI 1.08–7.94), mothers who received post-natal care (AOR = 4.34; 95% CI 2.37–7.94), and those desiring less number of children (AOR = 5; 95% CI 2.19–11.41) were more likely to use modern family planning methods during the extended postpartum period. Therefore, health care providers should work to improve quality of health services provided during antenatal care and postnatal care to enhance family planning utilization among post-partum women.

**Electronic supplementary material:**

The online version of this article (10.1186/s13104-018-3698-6) contains supplementary material, which is available to authorized users.

## Introduction

Fertility is one of the three principal components of population dynamics [1]. The majority of unintended pregnancies occur in developing countries accounting for more than one-third of all unintended pregnancies. Contraceptive use during the first year of postpartum period has the potential to avert many of these unintended pregnancies [2, 3]. This period is critical for two reasons: postpartum women have high needs for contraception, and these women have multiple contacts with the health facility either for postnatal or child immunization visits [4].

Pregnancies within the first 12 months after a birth or a birth to pregnancy interval of less than 12 months are at the highest risk for adverse health outcomes for both the mother and child. The 2012 London Summit on Family Planning highlighted that postpartum family planning has the potential in accelerating progress towards the Millennium Development Goals 4 and 5 [2, 5]. Post-partum family planning has been found to be crucial in minimizing the risks for adverse health outcomes among mothers and their children [6].

Factors that contribute to a postpartum women’s vulnerability to pregnancy include return of menses, less breastfeeding, return to sex, and the lack of contraception [7]. Women are at risk of becoming pregnant in the month before her first menstruation. Unfortunately, many women do not initiate family planning until menstruation has resumed [8, 9]. These are opportunities for reaching women with contraception services during post-partum period [10, 11]. Analysis of findings from a study conducted across 17 countries found that 40% of women in their first year of postpartum period reported that they plan to use a family planning method within 1 year after delivery, however, they are not doing so [12, 13].

In Ethiopia, while 37% of sexually active women were at a risk of pregnancy during the first 6 months of postpartum period. This risk increases to 64% among women 6–11 months postpartum, and then to 72% among women 12–23 months postpartum [9]. Although the majority of postpartum women indicate the desire to use contraceptives, contraceptive uptakes are often not offered to, or taken up by in first year of post-partum period [14]. This study therefore aims to assess the magnitude and determinants of contraceptive use in extended postpartum period. The study will help policy makers and health sector managers to pay attention to the issue, while also enabling women and their family’s to adopt and utilize modern contraceptive methods in the extended postpartum period.

## Main text

### Methods

The study employed a community based cross-sectional study in Gida Ayana district. Gida Ayana district is one of the 17 districts found in East Wollega zone of the Oromia regional state. Ayana town, the capital of the district, is located 444 km to the west of Addis Ababa and 112 km to the North of Nekemte, capital city of the zone. Administratively, the district is divided into 19 rural kebeles (subdistricts) and 6 towns. The total population of the district is 108,744, with postpartum woman comprising about 4.09%. All postpartum women aged 15–49 years (who gave births between 6 months and 1 year of extended postpartum period) prior to the study period and not pregnant were included in this study.

The source population were all women in the extended postpartum period (6–12 months). Sample size was calculated using the single population proportion formula based on the following assumptions: 24% postpartum family planning (PPFP) use in 6–12 months in Ethiopia [14], 5% margin of error, 95% confidence level (Z_α∕2_), 10% non-response rate and considering the design effect of two. The estimated sample size was thus 616.

A multi-stage sampling technique was used to enrol study participants. The district consists of 25 kebeles (19 rural and 6 urban) that comprise of 26,686 households. Out of these, 12 kebeles were selected using simple random sampling from both stratified samples (heterogeneous groups-urban and rural kebeles) then proportion to women’s who had live birth and were in their extended postpartum period (PPFP). Women in extended postpartum period were screened by initial enumeration through house to house survey. Then eligible households were selected by using systematic random sampling from the sampling frame of women in their extended postpartum period.

A structured questionnaire was developed based on literatures, and adapted to local situation [15, 16]. The questionnaire was prepared in English and translated into the local language, Afan Oromo, then back to English in order assure its reliability. The modern family planning methods were use of short acting methods (pills, condoms, injectables) or long acting methods (implants, IUD). The interview was administered in the Oromo language. The data collection was performed by 24 trained non-health professionals with at least a college diploma. A three days training was provided to them on the objective of the study. The questionnaire was pre-tested in Kiremu district to ensure consistency and reliability of data.

Data were entered, cleaned, and analyzed using SPSS version 20. Then a univariate analysis were conducted using frequency and percentage to summarize the data. Variables having p-value ≤ 0.2 in the bivariate analyses were fitted into a multiple logistic regression analysis to identify predictors of family planning use in the extended postpartum period. The model used forward stepwise multiple logistic regression technique to evaluate independent predictors of postpartum family planning (PPFP) by controlling the effect of others. The strength of association between family planning use in extended partum period and predictors was expressed in OR through a 95% confidence interval.

### Results

#### Socio-demographic and economic characteristics of the respondents

Six hundred three post-partum women completed the questionnaire making up a response rate of (97.9%). Majority of the respondents were from rural areas (68.2%). The mean age of participants was 26.9 years (SD ± 5.16). The majority (94.5%) of the respondents were married and more than half (54.7%) of the respondents were not able to read and write. Respondents who had a monthly income of < 500 ETB and 1000–2000 ETB accounted for 33.2% and 33.5% respectively. Of the total respondents, 38.5% have a television in their homes. A large proportion of participants (68.5%) also travel a distance of less than 1 h on foot to get health services from the nearby health facilities (Additional file 1: Table S1).

#### Reproductive health characteristics of respondents

Most of the respondents (58.9%) reported that they were married before the age of 18. A third (32.0%) of women gave birth to their first child before their 18th birthday. Nearly half (49.4%) of the women reported their desire to have more than four children. Most of the women (68.7%) did not know their fertile period after giving birth. The majority (92.2%) of the respondent mothers were already engaged in sexual activity after giving birth of a baby. A large proportion of respondents (87.6%) agreed that their relatives or husband support utilization of modern family planning methods following birth (Additional file 2: Table S2).

#### Utilization of maternal health service

The study indicated that women who had 4 or more ANC visits were (28.5%). Half of the women (49.8%) gave birth at a health facility. More than half (57.0%) of respondents were within 9–12 months of post-partum period. A third (31.8%) had post-natal visit within 7 days–6 weeks of postpartum period (Table 1).

**Table 1:** Maternal health services utilization, Gida Ayana district, East Wollega zone, Ethiopia 2015 (n = 603)

Characteristics	Categories	Frequency (%)
Received ANC visits (n = 603)	< 4 visits	320 (53.1%)
	4 and more visits	172 (28.5%)
	No visit	111 (18.4%)
Place of delivery (n = 603)	Home	303 (50.2%)
	Health facility	300 (49.8%)
Birth attended by (n = 603)	Skilled birth attendants	306 (50.7%)
	Non skilled birth attendants	297 (49.3%)
Month since last birth/since recent delivery (n = 603)	6–9 months	259 (43.0%)
	9–12 months	344 (57.0%
Timing of postnatal check-up (n = 603)	Within 7 days	104 (17.2%)
	Between 7 days and 6 weeks	192 (31.8%)
	After 6 weeks	130 (21.8%)
	No PNC	177 (29.4%)
Child condition (n = 603)	Alive	600 (99.5%)
	Dead	3 (0.5%)

Majority (53.6%) of the respondents had a birth interval of 24–36 months between the last two births. Majority (89.6%) of the mothers had exposure to family planning message during pregnancy and birth. Similarly, 92.5% agreed that using modern family planning methods within postpartum period is important, however, 45.4% of the respondents utilize some form of modern contraceptive methods. The most commonly used family planning methods were injectables and implants (54.7% and 28.7%), respectively. The main reason given by the mothers for not using modern family planning methods was their menses not returned after giving birth (36.0%) (Table 2).

**Table 2:** Percentage distribution of respondents by their obstetrics history and fertility, Gida Ayana district, East Wollega zone, Ethiopia 2015 (n = 603)

Characteristics	Categories	Frequency (%)
Birth interval in months (n = 603)	< 24 months	184 (30.5%)
	24–36 months	323 (53.6%)
	More than 36 months	96 (15.9%)
Exposure to family planning messages during pregnancy (n = 603)	Yes	540 (89.6%)
	No	63 (10.4%)
Accepting importance of using PPFP during 6–12 months of postpartum period? (n = 603)	Yes	558 (92.5%)
	No	45 (7.5%)
Used family planning method within 6–12 months of postpartum period (n = 603)	Yes	274 (45.4%)
	No	329 (54.6%)
Family planning methods used (n = 274)	Injectable	150 (54.7%)
	Implants	78 (28.5%)
	IUCD	40 (14.6%)
	Oral pills	6 (2.2%)
Reason for not using family planning methods within 6–12 months of post-natal period (n = 329)	Menses not returned	118 (36.0%)
	Using breast feeding	85 (25.8%)
	Fear of side effect	34 (10.3%)
	Want to have children	26 (7.9%)
	Has no partner/no sex	20 (6.1%)
	Did not want	17 (5.2%)
	Husband objection	11 (3.3%)
	Religious opposition	10 (3.0%)
	Long distance of the facility from home	8 (2.4%)

#### Factors associated with utilization of PPFP

According to results of the bi-variate analysis: residence, occupation, current age of the mother, knowledge about modern family planning methods, level of education, marital status, availability of radio and television, distance of health facilities from residence, average monthly income, discussion with the partner, knowledge of fertile period, number of ANC visit, place of delivery, timing of PNC checkup, months since last delivery, sexual contact after delivery, approval from the partner or relative to use family planning and the desire to have another child were found to be associated with utilization of family planning. Mothers who had 4 or more ANC visits (AOR 2.93 95% CI 1.08–7.94), mothers who received post-natal care (AOR 4.34; 95% CI 2.37–7.94) and those desiring lower number of children (AOR 5.0 95% CI 2.19–11.41) showed significant positive association with utilization of modern family planning methods during the extended postpartum period (Table 3).

**Table 3:** Predictors of utilization of PPFP methods among women who gave birth in the last 12 months in Gida Ayana district, 2015

Variables	Categories	Utilization of PPFP		COR (95% CI)	AOR (95% CI)
		Yes	No		
Received ANC visit	< 4 visit	151 (47.2%)	169 (52.8%)	*7.46 (3.9*–14.12)**	*7.14 (1.1*–50.00)*
	4 and more visit	111 (64.5%)	61 (35.5%)	*15.01 (7.63*–29.5)**	1.3 (1.15–3.95)*
	No visit	12 (10.8%)	99 (89.2%)	1	1
Sexual activity after last birth	Not yet started	7 (14.9%)	40 (85.1%)	1	1
	Engaged/resumed	267 (48.0%)	289 (52%)	*5.27 (2.32*–11.98)**	*5.55 (1.33*–23.12)*
Received postnatal care (visit)	Yes	220 (64.7%)	120 (35.3%)	*7.09 (4.88*–10.3)**	*4.34 (2.37*–7.94)**
	No	54 (20.5%)	209 (79.5%)	1	1
Desired number of children by the family	1–2 children	175 (68.4%)	81 (31.6%)	*6.58 (3.4*–12.7)**	*5.0 (2.19*–11.41)**
	3–4 children	67 (36.6%)	116 (63.4%)	*4.83 (3.36*–6.94)**	*5.19 (3.18*–8.47)**
	≥ 5 children	32 (19.5%)	132 (80.5%)	1	1
Residence	Urban	107 (55.7%)	85 (44.3%)	0.54 (0.38–0.76)**	1.17 (0.75–1.84)
	Rural	167 (40.6%)	244 (59.4%)	1	1
Knowledge of fertile period after delivery	Know fertile period	113 (59.8%)	76 (40.2%)	2.33 (1.64–3.32)**	1.06 (0.57–1.97)
	Doesn’t know	161 (38.9%	253 (61.1%)	1	1

1.0 = referent category, * statistically associated p value < 0.05, ** statistically associated p value < 0.001

### Discussion

This community-based survey assessed the utilization and factors associated with modern contraceptive methods use in the extended postpartum period. The findings found that about half of the study participants used modern family planning methods during the 6–12 months postpartum period. This finding is consistent with a study from Gonder, Ethiopia [8], while being slightly higher than studies done in 27 low and middle-income countries (35%) [13] as well as the Demographic and Health Survey re-analysis (37%) [14]. The observed discrepancy could be justified by the difference in study setting since the previous studies were conducted predominantly in rural areas. The finding showed a lower uptake of family planning during post-partum compared to a study from Malawi which reported a 75% uptake [5], and another study from Mekele, Ethiopia with a 61.3% uptake [10]. The difference might be due to factors related to access to health care facilities. This study largely covered the rural community in which the residents could easily get exposed to misconceptions and myths due to limited health literacy while the other studies were conducted primarly in urban settings where access and service utilization would be high. Postpartum modern contraceptive use was significantly associated with family planning counseling during ANC and PNC, utilization of PNC services, and resuming sexual activities.

The findings of this study showed that mothers who had four and more ANC visit were three times more likely to use PPFP compared to those who had no follow up at all. This is consistent with studies from Mekelle, Ethiopia [10] and Kenya [17]. Mothers who desire to have a lower number of children were more likely to use PPFP. Mothers who had postnatal care were more likely to practice modern PPFP methods. This could be due to the fact that mothers who attended postnatal care have an opportunity to communicate with providers and to receive counseling regarding postpartum modern family planning [3, 17, 18].

The reasons cited for not using post-partum modern family planning were: non-return of menstruation, assumptions of no risk, using breast-feeding, and fear of side effect. Similar reasons were identified in other studies [5, 8] (Additional file 3: Figure S1). This study provides an opportunity to address the high proportion of births within short intervals as well as improve maternal and child health outcomes.

In conclusion, the level of PPFP use was relatively low despite high levels of exposure to modern family planning messages and awareness on the importance of using modern contraceptive methods during 6–12 months of postpartum period among married women. Number of ANC visits, sexual contact after last delivery, received postnatal care (visit), and desired number of children by the family were the main predictors for the utilization of modern family planning methods during the postpartum period. This may suggest that having information on importance of PPFP methods during the extended period by itself is not sufficient to practice family planning.

Therefore empowering women, integrating maternal health services (such as ANC, delivery and PNC services) with modern family planning services, awareness creation on the risk of pregnancy during early and late postpartum period were highly suggested.

## Limitations

The primary limitation of the study was the use of a cross-sectional design, which precludes assessing the temporality and causality of the associations described. Recall bias might have been introduced on some of the questions that required the women to recall past information. Reporting inaccurate information to an interviewer in order to please him or her could also result in a social desirability bias. The data collectors were trained to minimize this bias.

## Additional files

**Additional file 1: Table S1.** Socio-demographic and economic characteristics of participants of Gida Ayana district, East Wollega zone, Ethiopia.

**Additional file 2: Table S2.** Reproductive characteristics of the study participants, Gida Ayana district, East Wollega zone, Ethiopia 2015.

**Additional file 3: Figure S1.** Reasons for not using modern family planning methods in the extended postpartum period GidaAyana district.

## Abbreviations

EDHS: Ethiopian Demographic and Health Survey
PPFP: postpartum family planning
CI: confidence interval
ANC: ante natal care
AOR: adjusted odds ratio
PNC: post natal care
SPSS: Statistical Package for Social Sciences
SD: standard deviation
ETB: Ethiopian Birr
