# Supplementary material for: Role of antenatal and postnatal care in contraceptive use during postpartum period in western Ethiopia: a cross sectional study
Source: BMC Res Notes. 2018 Aug 13;11:581. doi: 10.1186/s13104-018-3698-6 (PMC6090703; doi:10.1186/s13104-018-3698-6)
Supplement: Supplementary file 1 — Additional file 1: Table S1. Socio-demographic and economic characteristics of participants of Gida Ayana district, East Wollega zone, Ethiopia. [file 13104_2018_3698_MOESM1_ESM.docx]

Additional file 1: Table S1 Socio-demographic and economic characteristics of participants of Gida Ayana district, East Wollega Zone, Ethiopia 2015 (n = 603)

| Characteristics | Categories | Frequency (%) | Using FP | Not Using FP | chi square, df, p value |
| --- | --- | --- | --- | --- | --- |
| Place of residence(n=603) | Urban | 192(31.8%) | 107 (39.1%) | 85 (25.8%) | χ 2 =12.030  df =1 |
|  | Rural | 411(68.2%) | 167 (60.9%) | 244 (74.2%) | p-value = .001 |
| Age group(n=603) | 15-19 | 39(6.4%) | 22 (8.0%) | 17 (5.2%) | χ2 =41.764 |
|  | 20-24 | 155(25.7%) | 94 (34.3%) | 61 (18.5%) | df = 4 |
|  | 25-29 | 212(35.2%) | 102 (37.2%) | 110 (33.4%) | p-value = .000 |
|  | 30-34 | 136(22.5%) | 43 (15.7%) | 93 (28.3%) |  |
|  | >=35 | 61(10.2%) | 13 (4.7%) | 48 (14.6%) |  |
| Marital status(n=603) | Married | 570(94.5%) | 271(98.9%) | 299 (90.9% | χ2 =19.773 |
|  | Single | 16(2.7%) | 3 (1.1%) | 13 (4.0%) | df = 3 |
|  | Divorced | 8(1.3%) | 0 (.0%) | 8 (2.4%) | p-value = .000 |
|  | Separated | 7(1.2%) | 0 (.0%) | 9 (2.7%) |  |
|  | Widowed | 2(0.3%) |  |  |  |
| Educational level(n=603) | Illiterate | 330(54.7%) | 92 (33.6%) | 238 (72.3%) | χ2 =92.578a |
|  | Primary education | 178(29.5%) | 114 (41.6%) | 64 (19.5%) | df = 3 |
|  | Secondary education | 72(11.9%) | 53 (5.8%) | 19 (19.3%) | p-value = .000 |
|  | College and above | 23(3.8%) | 15(5.5%) | 8 (2.4%) |  |
| Mothers’ occupation(n=603) | Farmer | 364(60.4%) | 133(48.5%) | 231(70.2%) | χ2 = 62.154 |
|  | House wife | 152(25.2%) | 81(29.6%) | 71(21.6%) | df = 4 |
|  | GO/NGO employee | 35(5.8%) | 34(12.4%) | 1(.3%) | p-value = .000 |
|  | Private employee | 34(5.6%) | 12(4.4%) | 22(6.7%) |  |
|  | Student | 18(3.0%) | 14(5.1%) | 4 (1.2%) |  |
| Religion(n=603) | Orthodox | 247(41.0%) | 120 (43.8%) | 127 (38.6%) | χ2 = 1.814 |
|  | Muslim | 160(26.5%) | 71(25.9%) | 89 (27.1%) | df = 3 |
|  | Protestant | 189(31.3%) | 80 (29.2%) | 109 (33.1%) | p-value = .612 |
|  | Catholic | 7(1.2%) | 3 (1.1%) | 4 (1.2%) |  |
| Ethnicity(n=603) | Oromo | 348(57.7%) | 154(56.2%) | 194 (59.0%) | χ2 =4.820 |
|  | Amhara | 231(38.3%) | 106(38.7%) | 125 (38.0%) | df = 3 |
|  | Tigre | 22(3.6%) | 14(5.1%) | 8 (2.4%) | p-value = .185 |
|  | Gurage | 2(0.3%) | 0(.0%) | 2(.6%) |  |
| Monthly income(n=603) | <500Birr | 200(33.2%) | 47(17.4%) | 153(46.6%) | χ2 =63.954 |
|  | 500-1000Birr | 144(23.9%) | 68(25.2%) | 76(23.2%) | df = 3 |
|  | 1001-2000Birr | 202(33.5%) | 123(45.6%) | 79(24.1%) | p-value = .000 |
|  | >=2000 Birr | 52(8.6%) | 32(11.9%) | 20(6.1%) |  |
|  | No response | 5(0.8%) |  |  |  |
| Distance of health facility from residence on foot (hr)(n=603) | <1hour | 413(68.5%) | 216(78.8%) | 197(59.9%) | χ2 =45.152 |
|  | 1-2 hour | 148(24.5%) | 58(21.2%) | 90(27.4%) | df =2 |
|  | >2 hour | 42(7.0%) | 0(.0%) | 42(12.8%) | p-value =.000 |
