# Supplementary material for: Role of antenatal and postnatal care in contraceptive use during postpartum period in western Ethiopia: a cross sectional study
Source: BMC Res Notes. 2018 Aug 13;11:581. doi: 10.1186/s13104-018-3698-6 (PMC6090703; doi:10.1186/s13104-018-3698-6)
Supplement: Supplementary file 2 — Additional file 2: Table S2. Reproductive characteristics of the study participants, Gida Ayana district, East Wollega zone, Ethiopia 2015. [file 13104_2018_3698_MOESM2_ESM.docx]

Additional file 2: Table S2 Reproductive characteristics of the study participants, Gida Ayana district, East Wollega Zone, Ethiopia 2015 (n=603)

| Characteristics | Categories | Frequency (%) |
| --- | --- | --- |
| Age at marriage(n=603) | <18years | 355(58.9%) |
|  | >=18 years | 232(38.5%) |
|  | None | 16(2.7%) |
| Age at first birth(n=603) | <18 years | 193(32.0%) |
|  | ≥18 years | 410(68.0%) |
| Total children desired(n=603) | 1-2 children | 50(8.3%) |
|  | 3-4 children | 255(42.3%) |
|  | >4 children | 298(49.4%) |
| Decision maker on the number of children(n=603) | Both wife and husband | 466(77.3%) |
|  | Wife | 104(17.2%) |
|  | Husband and family | 33(5.5%) |
| Knowledge of fertile period after giving birth of a baby(n=603) | Know fertile period | 189(31.3%) |
|  | Does not know | 414(68.7%) |
| Current status of sexual activity  (n=603) | Abstaining/no sexual activity | 47(7.8%) |
|  | Engaged/resumed in sexual activity | 556(92.2%) |
| Opposition to using family planning(n=603) | Perceived opposition from family or relatives | 75(12.4%) |
|  | No opposition from family or community | 528(87.6%) |
