# Supplementary material for: Role of antenatal and postnatal care in contraceptive use during postpartum period in western Ethiopia: a cross sectional study
Source: BMC Res Notes. 2018 Aug 13;11:581. doi: 10.1186/s13104-018-3698-6 (PMC6090703; doi:10.1186/s13104-018-3698-6)
Supplement: Supplementary file 3 — Additional file 3: Figure S1. Reasons for not using modern family planning methods in the extended postpartum period GidaAyana district. [file 13104_2018_3698_MOESM3_ESM.docx]

Additional file 3: Figure S1. Reasons for not using modern family planning methods in the extended postpartum period GidaAyana district.
